# To My Anti

**Published:** 1898-07

**Authors:** 


### To My Anti.

What cures my head when full of aches,
All joy my racking brain forsakes,
And all life's pleasures seem but "fakes"?
My Anti.

What cools me when I feel so hot,
As Dives when below he got
And envied Lazarus' happy lot?
My Anti.

What helps me when I have the fits,
And every one who round me sits
Immediately gets up and "gits"?
My Anti.

What fills the German chemist's purse,
And incidentally perhaps the hearse?
What always terminates my verse?
My Anti.

